# Economic evaluation of an extended nutritional intervention in older Australian hospitalized patients: a randomized controlled trial

**DOI:** 10.1186/s12877-018-0736-0

**Published:** 2018-02-05

**Authors:** Yogesh Sharma, Campbell Thompson, Michelle Miller, Rashmi Shahi, Paul Hakendorf, Chris Horwood, Billingsley Kaambwa

**Affiliations:** 1 Department of General Medicine, Flinders Medical Centre, Adelaide, South Australia Australia; 2 College of Medicine and Public Health, Flinders University, Adelaide, South Australia Australia; 3 Discipline of Medicine, University of Adelaide, Adelaide, South Australia Australia; 4 Department of Nutrition & Dietetics, Flinders University, Adelaide, South Australia Australia; 5 Faculty of Health Sciences, Flinders University, Adelaide, South Australia Australia; 6 Department of Clinical Epidemiology, Flinders Medical Centre, Adelaide, South Australia Australia; 7 Health Economics Unit, Flinders University, Adelaide, South Australia Australia

**Keywords:** Malnutrition, Economic evaluation, Health related quality of life, Quality adjusted life years, Older patients

## Abstract

**Background:**

Prevalence of malnutrition in older hospitalized patients is 30%. Malnutrition is associated with poor clinical outcomes in terms of high morbidity and mortality and is costly for hospitals. Extended nutrition interventions improve clinical outcomes but limited studies have investigated whether these interventions are cost-effective.

**Methods:**

In this randomized controlled trial, 148 malnourished general medical patients ≥60 years were recruited and randomized to receive either an extended nutritional intervention or usual care. Nutrition intervention was individualized and started with 24 h of admission and was continued for 3 months post-discharge with a monthly telephone call whereas control patients received usual care. Nutrition status was confirmed by Patient generated subjective global assessment (PG-SGA) and health-related quality of life (HRQoL) was measured using EuroQoL 5D (EQ-5D-5 L) questionnaire at admission and at 3-months follow-up. A cost-effectiveness analysis was conducted for the primary outcome (incremental costs per unit improvement in PG-SGA) while a cost-utility analysis (CUA) was undertaken for the secondary outcome (incremental costs per quality adjusted life year (QALY) gained).

**Results:**

Nutrition status and HRQoL improved in intervention patients. Mean per included patient Australian Medicare costs were lower in intervention group compared to control arm (by $907) but these differences were not statistically significant (95% CI: -$2956 to $4854). The main drivers of higher costs in the control group were higher inpatient ($13,882 versus $13,134) and drug ($838 versus $601) costs. After adjusting outcomes for baseline differences and repeated measures, the intervention was more effective than the control with patients in this arm reporting QALYs gained that were higher by 0.0050 QALYs gained per patient (95% CI: -0.0079 to 0.0199). The probability of the intervention being cost-effective at willingness to pay values as low as $1000 per unit improvement in PG-SGA was > 98% while it was 78% at a willingness to pay $50,000 per QALY gained.

**Conclusion:**

This health economic analysis suggests that the use of extended nutritional intervention in older general medical patients is likely to be cost-effective in the Australian health care setting in terms of both primary and secondary outcomes.

**Trial registration:**

ACTRN No. 12614000833662. Registered 6 August 2014.

## Background

Malnutrition is common in older hospitalized patients with prevalence rates as high as 30% in acute care settings in Australia [1]. Malnutrition is associated with adverse clinical outcomes for patients in terms of higher morbidity and mortality [2] and is costly for the hospitals [3]. The adverse effects associated with malnutrition on patient outcome and recovery results in increased health care use and costs [4]. Health-care costs are increased because malnourished patients stay longer in hospitals, suffer more infectious and non-infectious nosocomial complications, experience frequent hospital re-admissions and have higher utilization of health-care resources in the community [5–8]. Three recent meta-analyses [9–11] have indicated that nutrition intervention has economic benefits but have also suggested that there is a need for further high quality studies to confirm these findings in different age groups and in different health care settings. This is especially so as majority of these studies have been conducted in Europe and very few studies are available in the Australian health care settings.

A recent randomized controlled trial [12] conducted in a large tertiary hospital in Australia from 2014 to 2016, assessed efficacy of an early and extended nutrition intervention in older hospitalized patients. In this trial, an individualized nutrition intervention was started within 24 h of hospital admission and patients ≥60 years age received monthly telehealth follow up for two months following discharge and this intervention was compared to usual care. The main objectives in this trial was to examine whether such an intervention could improve nutritional status and quality of care by reducing adverse clinical outcomes and optimizing use of existing resources.

This trial found a trend towards an improvement in nutritional status and quality of life and a significant reduction in length of hospital stay but there was no reduction in mortality or readmissions at 3 months follow up. Although the resources needed for the intervention were modest and the anticipated improvement in the nutrition status was small [12], no economic evaluation was conducted to examine whether the intervention was worth pursuing from an economic perspective. The objective of the present analysis was to conduct an economic evaluation that assessed whether the individualized nutrition intervention was value for money when considered from a healthcare sector (Australian Medicare) perspective. The results of the evaluation will help determine whether allocation of resources for improvement of nutritional status of older hospitalized patients is justifiable. Consequently, the primary outcome of this evaluation was expressed in terms of incremental costs per unit improvement in the PG-SGA (CEA) and the secondary outcome reported in terms of incremental costs per QALY gained (CUA).

## Methods

### Study design

The data for this health economic analysis were obtained from a recently conducted nutrition intervention study [12], which was designed as a randomized controlled trial.

### Target population

The participants for this study included hospitalized patients aged ≥60 years, who were confirmed as malnourished by a qualified dietitian using PG-SGA tool [13].

### Sample size

The sample size was calculated based upon the change in the PG-SGA score from the baseline in the clinical trial [12] which provided data for this economic evaluation. The sample size in the clinical trial was based on the findings of a previous study [14], which has suggested that a shift of 3 (SD 4.1) in PG-SGA is clinically meaningful, assuming an affect size of 0.35, alpha = 0.05 and power of 80% the estimated sample size was 86 (43 in each group) was calculated to be sufficient.

### Setting and location

This study included patients presenting to the Department of General Medicine, Flinders Medical Centre (FMC), Adelaide, South Australia. FMC is a tertiary level, teaching hospital with 520 beds capacity and the Department of General Medicine admits approximately 4500 patients per year. Health services at FMC are predominantly funded through the Australian Medicare Scheme (the primary funder of universal healthcare insurance in Australia). Patients were excluded if they were receiving palliative care, residing in rural areas, or were of indigenous origin or were non-English speaking. Rural, indigenous and non-English speaking subjects were excluded due to lack of funds to travel to rural areas for assessments and seek services of an Indigenous liaison officer/interpreter.

### Study perspective

The direct costs of implementing nutritional intervention were determined from the Australian (Medicare) health care perspective. These included costs of hospitalizations, dietitian costs for post-discharge telephone calls, costs of providing nutrition supplements, post-discharge general practioner (GP) and specialist physician visits. Other costs were for any outpatient investigations and procedures, allied health care utilization and medicinal products over the period of 3-months of intervention. Indirect costs, such as those incurred by the patients due to loss of productivity were not included in this analysis.

### Comparators

The economic evaluation determined the relative cost-effectiveness/cost-utility of the intervention when compared to the control.

#### Intervention

Nutrition intervention was initiated within 24 h of hospital admission and aimed to meet 100% of patients’ energy and protein requirements for ideal body weight, calculated using commonly adopted predictive equations [15] along with an adequate intake of essential vitamins and minerals. Intervention patients received an individualized nutrition intervention by the dietitian, depending upon their underlying medical conditions, protein, energy, vitamin and mineral requirements and food preferences. Nutritional strategies employed by the dietitian included provision of oral nutrition supplements (ONS) (1–2.2 kcal/ml and 0.05–0.12 g of protein/ml), mid-meal snacks and food fortification with consideration given to individual patients’ food preferences and taste. The ONS utilized were Resource (Nestle Heath Science) (475 kcal, 19.7 g protein) and Sustagen (Nestle Heath Science) (248 kcal, 12.5 g protein), which in addition to protein provided a range of nutrients. Multivitamins were not separately prescribed but were left to the discretion of the treating clinicians. In addition, the patients and their care-providers received dietetic counseling, to augment their energy intake by using a range of strategies including recommendation of energy and nutrient dense food items, increasing the number of meals they ate, and consumption of energy, protein and nutrient-rich snacks. Patients who needed assistance with meals were flagged, so that a ward based staff member provided help during meals. The frequency of contact between patient and dietitian during the hospital stay varied depending upon individual patients’ needs and the length of hospital stay. If the dietitian thought that the patient was unable to achieve their daily energy and nutrient requirements then they received almost daily input. Where patients were discharged to a nursing home then the dietitian contacted the nursing home manager and forwarded the recommended nutritional care plan to be followed. The hospital covered the cost of commercial oral nutritional supplements at the time of discharge for patients where ≥50% of the patient’s daily energy requirements were determined to be required from supplements. All intervention patients were contacted by a monthly telephone call by the research dietitian for 2 months. During this interview, a structured format was used by the dietitian to collect information about patients’ recent weight, compliance with the dietetic plan and any side effects with supplementation. In addition, patients received dietetic counseling with a focus to reinforce compliance with the intervention. Compliance with the dietetic plan was assessed by using a 24-h self-reported dietary recall. In this trial, the dietitian assessed the patients as compliant to the nutritional care plan if they were able to meet at least 75% of their energy and protein requirements.

#### Control group

Patients randomized to the control group followed usual care currently operative in Flinders Medical Centre. Currently all patients undergo nutrition screening by the use of Malnutrition Universal Screening Tool (MUST) and patients identified as high risk for malnutrition are referred to the dietitian. However, dietetic input occurs only if clinicians refer the patients and even if a dietitian sees them during hospital admission, they may not be followed after discharge. In this study, the control patients were flagged as malnourished and this was documented in the case notes for clinicians to make decisions regarding nutritional care. If control patients got referred for a dietetic advice, then they were offered the same nutritional care plan as the intervention group only for the period of their hospitalization but received no post discharge follow up care.

### Time horizon

The costs between the two groups were compared over a period of three months from the time of randomization during hospital admission until the last follow-up.

### Discount rates

Discounting of costs and effectiveness measures was not performed, because the time horizon of this study did not exceed 1 year [16].

### Choice of nutritional/health outcomes

The primary nutritional outcome in this study, as was the case in the clinical study [12] and for the sake of maintaining consistency, was the unit improvement in the PG-SGA over the 3-month study period. The secondary outcome was QALYs gained over the same period and based on the responses to the EuroQoL 5 Dimensions 5 Levels (EQ-5D-5 L) [17].

#### PG-SGA

The nutrition status of the participants was confirmed with PG-SGA by an experienced dietitian. The PG-SGA [18] generates a numerical score while also providing an overall global rating divided into three categories: well nourished (PG-SGA A), moderately malnourished or suspected of being malnourished (PG-SGA B) or severely malnourished (PG-SGA C). For each component of the PG-SGA, points (0–4) are awarded depending on the impact on nutritional status. Component scores are summed up to obtain total scores that range from 0 to 35 and scores ≥7 indicating a critical need for nutritional intervention and symptom management in the older patients [19]. PG-SGA has been validated in various settings including older hospitalized patients and has a high sensitivity and specificity to diagnose malnutrition [19].

#### HRQoL and QALYs

QALYs gained were chosen as an outcome as they facilitate comparisons between interventions for disparate services and are recommended for use by decision makers including the Pharmaceutical Benefits Advisory Committee (PBAC) in Australia [20].

QALY estimates, calculated using the area-under-the-curve method [16], were based on responses to the EQ-5D-5 L which were scored using UK value sets [17].

The EQ-5D-5 L is a self-reported questionnaire and measures a patient’s health across five different domains: mobility, self-care, usual activities, pain/discomfort and anxiety/depression [21]. Using these responses, the EQ-5D-5 L is able to distinguish between 3125 states of health. A UK-specific algorithm developed using time-trade-off techniques was used to convert the EQ-5D-5 L health description into a valuation ranging from − 0.281 to 1 [17]. Scores less than 0 represent health states that are worse than death [22]. The EQ-5D-5 L has been validated in different clinical populations including patients with multiple chronic illnesses, rehabilitation and orthopedic patients awaiting joint replacement surgery and has been found to have a stronger convergent validity coefficient (Spearman’s coefficient 0.51–0.75) and a higher absolute informativity (Shannon’s index) as compared to the EuroQol 5 Dimensions 3 Levels (EQ-5D-3 L) [23–25].

### Measurement of effectiveness

No effectiveness data were obtained from secondary sources as our analysis relied upon data from our original trial [12].

### Estimating resources and cost

Data on the volume and total costs of healthcare utilisation, measured from the health care perspective, were readily provided by Medicare Australia. Cost data were provided in the form of Medicare Benefits Schedule (MBS) data (number and costs of GP visits, specialist attendances, non-specialist attendance, diagnostic procedures and other medical services such as pathology and teleheath services); Pharmaceutical Benefits Schedule (PBS) data (quantity and costs of pharmaceuticals) and; centralised costing (Australian refined diagnosis related group (AR-DRG)) data [26] (number and costs of public hospital inpatient episodes). Patient consent was sought before obtaining MBS, PBS and AR-DRG data. Costs associated with the intervention itself (primarily dietitian staff costs for making follow-up telephone calls (30 min per month for two months i.e. two phone calls per patient for all patients) and costs of supplements for the entire study period for nearly half (36) of the patients) were estimated by combining staff time spent/number of supplements provided and published information on wage rates obtained from published resources ($37.16 per hour for an accredited dietitian) and unit costs for supplements sourced from hospital accounts records ($6 per package per day). All costs are reported in Australian dollars at 2016/17 unit prices [16].

### Analytical methods

#### Descriptive statistics

Continuous variables were expressed as mean (standard deviation (SD)) values or median (interquartile (IQR)) ranges and were compared using an appropriate parametric (Student t) test or nonparametric (Mann-Whitney U) test. Categorical variables were expressed as frequencies and percentages and were compared using Chi-square (χ^2^) statistics or Fishers exact test as appropriate. Length of hospital stay (LOS) was adjusted for in-hospital mortality.

#### Economic evaluation

Two types of economic evaluation (CEA and CUA) were used in this study. Their choice was informed by the types of outcomes measured in the main trial [12]. CEA is a type of economic evaluation whose outcomes are expressed in terms of natural units such as life expectancy or change in PG-SGA scores, while outcomes in CUA are reported in terms of QALYs [27]. Consequently, the primary outcome of this evaluation was expressed in terms of incremental costs per unit improvement in PG-SGA (CEA) and the secondary outcome reported in terms of incremental costs per QALYs gained (CUA). An incremental approach was used in order to determine, where appropriate, the incremental cost effectiveness ratios (ICERs) expressed as the incremental cost per unit improvement in the PG-SGA (primary outcome) and incremental costs per quality adjusted life year QALY gained (secondary outcome). The ICERs were calculated as incremental costs divided by incremental changes in outcomes. The economic evaluation was conducted using an intention-to-treat approach.

Within-trial economic evaluation with respect to the primary and secondary outcomes was undertaken allowing for bivariate uncertainty with bootstrapping of participant costs and outcomes to maintain the covariance structure. To account for uncertainty due to sampling variation in cost-effectiveness/cost-utility, non-parametric bootstrapping [28] were applied on participant level data to derive 5000 paired estimates of mean differences in costs and outcomes. These bootstrapped pairs were summarized within cost effectiveness planes (CEPs) [29]. The probability of the intervention being more cost effective, compared to the usual care arm at different willingness-to-pay thresholds, was depicted using Cost effectiveness acceptability curves (CEACs).

Due to the presence of missing data on costs and outcomes (Tables 1, 2 and 3), multiple imputation was used to account for missing values prior to conducting the base-case economic evaluation [30]. Imputed values were generated by use of an iterative Markov chain Monte Carlo method premised on multivariate normal regression. To appropriately characterize the uncertainty about the right value to impute, each missing value in the dataset was replaced with a set of 50 plausible values. Standard complete-case procedures were then applied to each of the 50 resultant multiply imputed datasets before combining the results using Rubin’s rules [31]. The following variables were used to predict missing values in the imputation procedure: study arm, age, gender, cognitive status, length of stay, total number of comorbidities and malnutrition diagnosis. In both the base-case and sensitivity analyses, only adjusted outcomes (adjusted for baseline differences and correlation between repeated measurements) were used.

**Table 1:** Baseline characteristics of participants

Characteristics	Control (*n* = 70)	Intervention (*n* = 78)	*P* value
Age, mean (95% CI), y	81.6 (79.5 to 83.6)	82.0 (80.0 to 83.9)	0.76
Gender, n (%)			
Male	23 (32.9)	31 (39.7)	
Female	47 (67.1)	47 (60.3)	0.38
Residence before admission, n (%)			
Home	66 (94.3)	68 (87.2)	0.11
Nursing Home	4 (5.7)	10 12.8)	
Cognition, n (%)			
Normal	67 (95.7)	74 (94.9)	0.56
Impaired	3 (4.3)	4 (5.1)	
No of co-morbidities, mean (95% CI)	6.3 (5.6 to 6.9)	6.1 (5.5 to 6.6)	0.64
Charlson index, mean (95% CI)	2.3 (1.9 to 2.8)	2.2 (1.8 to 2.7)	0.82
Medications at admission, mean (95% CI)	10.1 (9.0 to 11.2)	8.8 (7.8 to 9.7)	0.07
Principal diagnosis at admission, n (%)			
Respiratory	29 (41.4)	20 (25.6)	0.30
Cardiovascular	8 (11.4)	14 (18.0)	
Falls	10 (14.3)	13 (16.7)	
CNS	3 (4.3)	6 (7.7)	
Miscellaneous	20 (28.6)	25 (32.1)	
BMI, mean (95% CI), kg/m2	21.8 (20.7 to 22.8)	20.6 (19.7 to 21.5)	0.09
PG-SGA score, mean (95% CI)	13.3 (12.2 to 14.5)	12.1 (11.0 to 13.2)	0.11
EQ-5D-5 L index	0.6746 (0.617 to 0.729)	0.6934 (0.638 to 0.746)	0.62

Abbreviations: *CI* Confidence Interval, *CNS* Central Nervous System, *BMI* Body Mass Index *PG-SGA,* Patient Generated Subjective Global Assessment, *EQ-5D-5 L* EuroQol 5 Dimensions 5 Levels

**Table 2:** Mean costs per patient (AU $)

Costs^a^	Control		Intervention		Difference (Bootstrapped 95% CI)
Base Case Analysis (imputed cases)^b^	n	Mean	n	Mean	
3 month MBS costs					
GP Costs	70	347 (38)	78	311 (32)	−37 (− 134, 59)
Specialist Attendance Costs	70	20 (5)	78	12	− 7 (− 19, 4)
Non-Specialist Attendance Costs	70	251 (43)	78	243 (36)	− 8 (− 122, 100)
Diagnostic Procedures costs	70	200 (40)	78	197 (31)	−4 (−111, 94)
Other Medical Service costs^c^	70	396	78	253 (34)	− 143 (− 291, 2)
Total MBS costs	70	1216 (128)	78	1008 (97)	− 208 (− 529, 149)
3 month PBS costs					
Total drug costs	70	838 (186)	78	601 (57)	− 237 (− 703, 47)
3 month Inpatient (DRG) costs					
Total DRG costs	70	13,882 (1390)	78	13,134 (1439)	− 748 (− 4584, 3310)
Intervention costs					
Total intervention costs	70	0	78	286 (30)	286 (225, 352)
Total Costs	70	15,936 (1397)	78	15,029 (1430)	−907 (− 4854, 2956)
Sensitivity analysis (complete cases)^d^					
3 month MBS costs					
GP Costs	62	348 (43)	65	307 (39)	−41 (− 151, 92)
Specialist Attendance Costs	62	21	65	13	−9 (− 20, 8)
Non-Specialist Attendance Costs	62	247 (48)	65	251 (48)	4 (−108, 142)
Diagnostic Procedures costs	62	200 (42)	65	211 (39)	10 (−108, 121)
Other Medical Service costs^c^	62	389 (73)	65	248 (47)	−141 (− 334, 5)
Total MBS costs	62	1205 (143)	65	1029 (132)	−176 (− 495, 226)
3 month PBS costs					
Total drug costs	59	855 (217)	65	610 (65)	− 245 (− 832, 99)
3 month Inpatient (DRG) costs					
Total DRG costs	70	13,882 (1390)	78	13,134 (1439)	−748 (−3310, 4584)
Intervention costs					
Total intervention costs	70	0	78	286 (30)	286 (225, 352)
Total Costs	59	17,024 (1595)	60	12,078 (917)	−4947 (−9030, −1451)

^a^*MBS* Medicare Benefits Schedule, *PBS* Pharmaceutical Benefits Schedule, *DRG* Australian Refined Diagnosis Related Groups (AR-DRGs) cost weights used to cost hospital admissions, *GP* General Practioner, *Total costs* = MBS costs + PBS costs + DRG costs + Intervention costs

^b^Multiply imputed values. Multiple imputations carried out to account for up to 29 or 19% missing data on cost estimates

^c^Examples of other medical costs include pathology and telehealth services as well as allied-health care attendances

^d^Analysis restricted to non-missing total cost estimates (119 or 81%)

**Table 3:** Outcomes of study

Outcomes^a^		Control		Intervention	Difference (Bootstrapped 95% CI)
	n	Mean	n	Mean	
Base Case Analysis (imputed cases)^b^					
EQ-5D-5 L and QALY gains					
EQ-5D-5 L at baseline	70	0.6746 (0.0284)	78	0.6934 (0.0276)	0.1088 (−0.0489, 0.0916)
EQ-5D-5 L at 3 months	70	0.5787 (0.0407)	78	0.6358 (0.0349)	0.0571 (−0.0556,0.1560)
Unadjusted QALYs	70	0.1578 (0.0064)	78	0.1659 (0.0067)	0.0081 (−0.0090, 0.0265)
Adjusted^c^ QALYs					0.005 (−0.0079, 0.0199)
PG-SGA Scores					
PG-SGA Scores at baseline	70	13.3286 (0.5817)	78	12.1123 (0.4951)	−1.2163 (− 2.6163,0.1793)
PG-SGA Scores at 3 months	70	7.3770 (0.4098)	78	5.9136 (0.4054)	−1.4634 (− 2.4801, −0.1896)
Unadjusted improvement in PG-SGA Scores^d^	70	5.9516 (0.6594)	78	6.1987 (0.5547)	0.2471 (−1.4931, 1.8661)
Adjusted^c^ improvement in PG-SGA Scores^d^					1.3238 (0.0240, 2.3858)
Inpatient stay					
LOS in days	69	9.9 (7.2)	71	6.9 (5.3)	3.0 (0.9, 5.1)
Sensitivity analysis (complete cases)^e^					
EQ-5D-5 L and QALY gains					
EQ-5D-5 L at baseline	69	0.6736 (0.0290)	77	0.6926 (0.0272)	0.0189 (−0.0537, 0.1003)
EQ-5D-5 L at 3 months	60	0.5672 (0.0487)	69	0.6360 (0.0407)	0.0688 (−0.0553, 0.2043)
Unadjusted QALYs	59	(0.1553 (0.0076)	69	0.1658 (0.0075)	0.0105 (−0.0096, 0.0291)
Adjusted^c^ QALYs					0.0060 (−0.0086, 0.0216)
PG-SGA Scores					
PG-SGA Scores at baseline	69	13.3478 (0.5848)	74	12.0946 (0.5240)	−1.2532 (−2.9491, 0.2727)
PG-SGA Scores at 3 months	46	6.9783 (0.6167)	57	5.8070 (0.5185)	−1.712 (−2.7446, 0.3698)
Unadjusted improvement in PG-SGA Scores^d^	46	6.1739 (0.8876)	57	5.8596 (0.7081)	−0.3143 (− 2.4223, 1.8485)
Adjusted^c^ improvement in PG-SGA Scores^d^					0.9849 (−0.5601, 2.5912)

^a^*EQ-5D-5 L* EuroQoL 5 Dimensions 5 Levels, *QALY* Quality Adjusted Life Years, *PG-SGA* Patient Generated Subjective Global Assessment, *LOS* Length of Hospital Stay

^b^Multiply imputed values. Multiple imputations carried out to account for up to 12% of the EQ-5D-5 L utility scores (2 or 1% of baseline and 19 or 1% of 3-month EQ-5D-5 L scores)

^c^These scores have been adjusted for baseline differences

^d^These PG-SGA scores were reverse scored so that a positive score reflects an improvement in nutrition status

^e^Trial participants with complete information on baseline and 3-month outcomes

Sensitivity analyses were carried out to test the robustness of the base case results and they focused on evaluating the effect of missing cost and outcome data values on the economic evaluation results (i.e. comparing results based on complete cases and those estimated using multiple imputed values). All analyses were conducted in Microsoft Excel (2010) and Stata version 14.1.

## Results

### Descriptive statistics

**Fig. 1:**
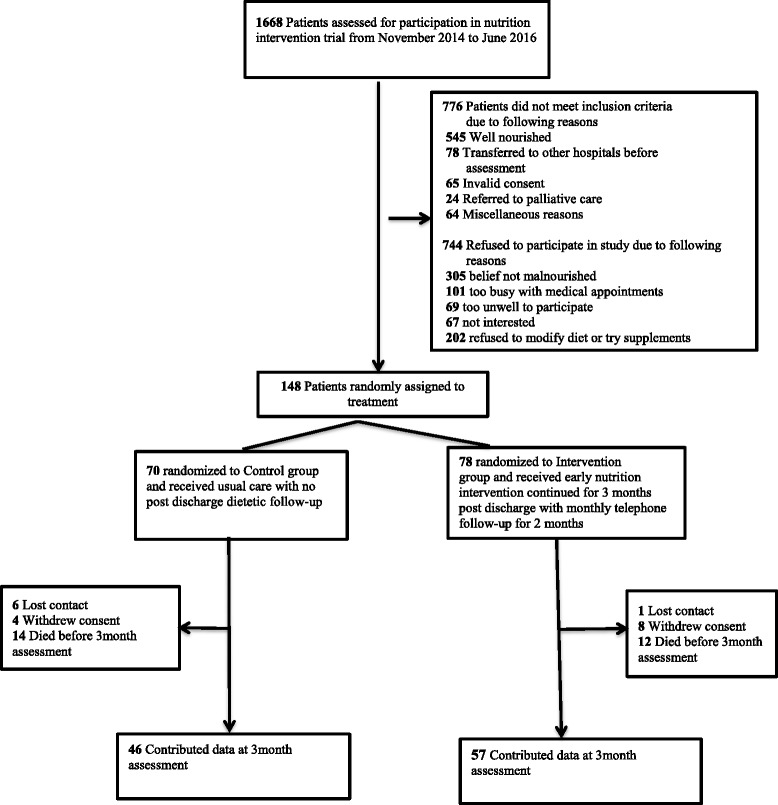
Study flow diagram

A total of 1668 patients (Fig. 1) admitted to the Department of General Medicine were assessed for participation in this study, whereof 892 met the inclusion criteria. Of the 892, 744 patients refused to participate due to various reasons (Fig. 1). One hundred and forty eight patients were therefore recruited and randomized to the control (*n* = 70) and intervention (*n* = 78) groups. The baseline clinical characteristics (Table 1) were similar between the two groups with regard to age, gender distribution, Charlson comorbidity index, number of medications and principal clinical diagnosis. There was no difference in severity of malnutrition at baseline as determined by PG-SGA score and HRQoL as determined by EQ-5D-5 L was similar between the two groups (Table 1). Nutritional intervention provided an additional mean 655 (95% CI: 587.3 to 772.1) kcal of energy and 36.5 (95% CI: 31.5 to 41.5) grams of protein and 73% and 77.2% patients were compliant with the intervention at 1 month and 2 months post-discharge, respectively. Length of hospital stay (LOS) was significantly shorter in the intervention patients (9.9 (SD: 7.2)) vs. 6.9 (SD: 5.3), *P* < 0.005) days, in control and intervention groups, respectively (Table 3).

### Incremental costs and outcomes

#### Base case analysis results

Table 2 presents a breakdown of mean healthcare costs per participant over a 3 months follow-up period. In the base case, mean per participant total Australian Medicare costs were lower in the intervention group compared to the control arm (by $907 per patient) but these differences were not statistically significant (95% CI: -$2956 to $4854). The main drivers of the higher costs in the control group were higher inpatient ($13,882 versus $13,134) and drug ($838 versus $601) costs. When the adjusted outcomes in the base case were considered (Table 3), the intervention was more effective than the control with participants in this arm reporting unit improvements in the PG-SGA that were higher by 1.3238 units (95% CI: 0.0240 to 2.3858) and QALYs that were higher by 0.0050 QALYs gained per patient (95% CI: -0.0079 to 0.0199). In line with best practice guidelines [32, 33], ICERs relating to both the primary and secondary outcomes are not presented, as the intervention was both cheaper and more effective regardless of outcome considered.

**Fig. 2:**
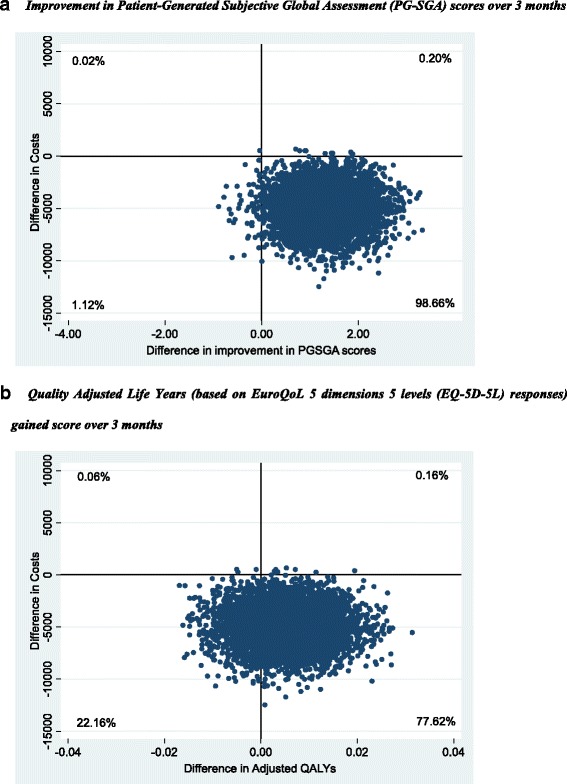
Cost-Effectiveness Planes

The CEPs in the base case analysis (Fig. 2) shows some uncertainly in the cost-effectiveness results but most of the bootstrapped paired estimates of mean differences in costs and outcomes appear in south-east and south-west quadrants.

**Fig. 3:**
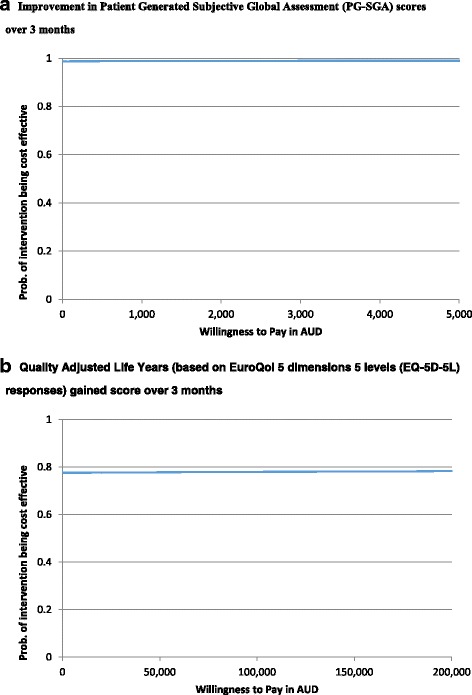
Cost-Effectiveness Acceptability Curves

The CEACs (Fig. 3) show that the probability of the intervention being cost-effective at willingness to pay values as low as $1000 per unit improvement in PG-SGA scores was above 98% while it was 78% at a willingness to pay of $50,000 per QALY gained, the implicit cost-effectiveness threshold used in Australia [34].

#### Sensitivity analysis results

In the base case analysis, multiple imputation was used to deal with the missing data on costs (29 observations or 20%), PG-SGA scores (45 observations or 30%) and EQ-5D-5 L responses (19 observations or 13%). In the sensitivity analysis, ignoring the missing data and using complete case analysis (Tables 2 and 3) did not have an effect on the incremental effectiveness. This is because the intervention was still more effective by 0.9849 units of improvement in the PG-SGA score (95% CI: -0.5601 to 2.5912) and by 0.0060 QALYs gained per patient (95% CI: -0.0086 to 0.0216), but was even more cheaper per patient (by $4947, 95% CI: $1451 to $9030). These figures did not change the final interpretation because the intervention still outperformed the control.

## Discussion

The findings of this study indicate that, in older general medical malnourished patients, the health care costs were lower while nutrition status and HRQoL was better among those in the individualized nutrition intervention arm compared to those in the group that received usual care with no post discharge dietetic follow-up. The differences in costs and HRQoL outcomes were however not statistically significant. In line with best practice guidelines [35, 36], therefore, our analysis focused on determining the likelihood of the intervention being cost-effective as opposed to hypothesis testing relating to whether the cost and QALY differences were statistically significant. Our results show that probability of the intervention being cost-effective at willingness to pay values as low as $1000 per unit improvement in PG-SGA was > 98% while it was 78% at a willingness to pay $50,000 per QALY gained. One of the strengths of this study is the use of PG-SGA for nutritional assessment, which has been demonstrated to have high sensitivity and specificity for the diagnosis of malnutrition and has been recommended as a predictive tool for clinical outcomes [14]. Yet, very few costing studies have utilized this stool for nutritional assessment.

At least two reasons may explain the statistically insignificant cost and HRQoL differences between the two trial arms. The first may be because the original trial [12] from which the data for this study were obtained was not powered to detect differences in costs and HRQoL, a result seen elsewhere [35, 36]. Another reason specific to HRQoL could be a short duration of nutrition intervention in our study. The impact of nutrition intervention on utilities is complex and may not be evident after a short period of intervention. After initiating nutrition intervention the temporal pattern that usually follows is - first improvement in nutrition parameters like weight then functional outcomes and lastly improvement in HRQoL [37]. Future nutrition intervention trials of sufficiently long duration may help verify this hypothesis.

The intervention was shown to have had lower mean Medicare costs than the control.

The cost drivers for the higher mean costs per patient in the control group were higher inpatient and drug costs. This could be related to the overall significantly longer length of hospital stay for the control patients with resultant higher utilization of health care resources. Studies have suggested that malnutrition contributes to the development of new complications such as delirium [38], predisposes to pressure ulcers [39] and increases risk of falls [40], all of which may contribute to the prolongation of the duration of hospitalization. Early nutrition intervention on the other hand may quickly improve the protein status and hence muscle function [41] as reflected by an increase in handgrip strength [42] and may lessen the risk of hospital acquired infections and may contribute to faster resolution of delirium [43]. It is possible that extension of this intervention following hospital discharge was associated with a sustained improvement in the nutrition status of intervention patients with a consequent reduction in the ‘post-hospital syndrome’ [44]. This may have led to a reduction in the utilization of primary health care resources (e.g. reduced GP visits) with consequent reduction in overall costs.

Our results are in line with a meta-analysis by Russell et al. [3] who found that use of ONS in surgical and elderly medical patients both in hospital and community settings can reduce LOS and complications with resultant net cost savings per patient. Our study is different from the studies used in the above meta-analysis in that we used a nutritional intervention tailored to individual patients needs rather than ONS alone, as studies have suggested poor compliance with ONS [45], especially in the older population. Similarly Gianotti et al. [46] found reduced treatment costs in patients who received enteral nutrition among patients undergoing major abdominal or cancer surgery and hypothesized that nutrition therapy helps improve splanchnic microperfusion with resultant lesser number of post-operative complications but in contrast to our study, this study included only surgical patients and limited nutrition intervention to the perioperative period. Norman et al. [47] in their study in malnourished patients aged 50.6 ± 16.1 years, with benign gastrointestinal disease found that 3-month nutritional supplementation with ONS increased HRQoL and was cost-effective from a German statutory health insurance perspective. Unlike our study, which included older patients with multiple comorbidities, however, this study was restricted to a relatively younger population of patients with benign gastrointestinal disease and nutrition intervention commenced only at the time of discharge. Our study results are also in line with the findings of three recent meta-analyses conducted in different patient groups [9–11], which suggest that the use of enteral medical nutrition in the management of disease related malnutrition (DRM) can be an efficient intervention from a health economic perspective and may lead to cost-savings.

Although malnutrition is common in older hospitalized patients, it is often poorly recognized by the clinicians with resultant fewer malnourished patients receiving treatment [48]. Economic evaluation offers a framework within which complex changes can be synthesized to aid in policy making. Our finding suggest that if similar intervention were to be delivered to all malnourished patients ≥60 years of age in General Medical service of our hospital in 2015–16, a per-patient cost saving of $907 will translate to a total savings of $1.86 million and if applied to the State of South Australia total cost savings of $9.05 million can be achieved. This study suggests that there is an opportunity to improve the health of malnourished older patients at a low marginal cost. Very few interventions have achieved health gains in this population at a lower cost [49]. In the current climate of economic constraints in healthcare, this study provides convincing evidence of the economic benefits of nutrition intervention.

### Limitations of study

Although the use of a randomized controlled study provides robust evidence for assessing the utility of nutrition intervention, this study had limitations when assessing economic value. Our analysis did not consider several factors, which could bias the results by either underestimating or overestimating the cost-effectiveness of nutritional supplementation. While we included the direct medical costs, we did not consider broader or indirect costs such as those borne by patients and their families privately or by nursing homes and costs associated with loss of work due to periods of absence for patients or their carers. Additionally, our study duration is limited to 3 months and long-term impact of such a nutrition intervention is unknown. Our study did have missing data on some costs and outcomes, however principled and robust methods were used to deal with the missingness. Finally, the difference in QALY gains in this study can be considered to be small and therefore our result on the effectiveness should be interpreted with caution. The overall economic evaluation results nevertheless considered these QALY gains jointly with cost differences as is appropriate.

Due to differences in design and organization of health-care systems, our study results cannot be generalized to other settings and countries and further studies are needed to contribute to the evidence of cost utility of nutritional therapy.

### Implications

Our study adds clinical and economic evidence of the benefits of initiating an early nutrition intervention with continuation in the community to improve health outcomes in older hospitalized malnourished population and justifies allocation of resources to improve the nutrition status of an elderly population.

## Conclusion

For both primary (change in PG-SGA scores) and secondary outcomes (QALY gains), the results of our health economic analysis suggest that the use of early and extended nutritional intervention in older general medical patients is likely to be cost-effective in the Australian health care setting as the intervention was both cheaper and more effective than the comparator. This conclusion was supported further by results of the CEACs that showed that the intervention had a high likelihood of being the cost-effective option over a range of willingness to pay values.

## Abbreviations

AR-DRG: Australian refined diagnosis related group
BMI: Body mass index
CEA: Cost effectiveness analysis
CEAC: Cost effectiveness acceptability curve
CEP: Cost effectiveness plane
CUA: Cost utility analysis
DRM: Disease related malnutrition
EQ-5D-5 L: European quality of life 5 dimension 5 level
HRQoL: Health related quality of life
LOS: Length of hospital stay
MBS: Medicare benefits schedule
MUST: Malnutrition universal screening tool
ONS: Oral nutritional supplements
PBAC: Pharmaceutical benefit advisory committee
PBS: Pharmaceutical benefits schedule
PG-SGA: Patient generated subjective global assessment
QALY: Quality adjusted life year
